# Implementation of flash glucose monitoring in four pediatric diabetes clinics: controlled before and after study to produce real-world evidence of patient benefit

**DOI:** 10.1136/bmjdrc-2023-003561

**Published:** 2023-08-28

**Authors:** Rebecca Kandiyali, Hazel Taylor, Elizabeth Thomas, Freyja Cullen, William Hollingworth, Jenny Ingram, Charlie Kenward, Nicol West, David McGregor, Becky Smith, Julian Hamilton-Shield

**Affiliations:** 1 Centre for Health Economics, Warwick Medical School, Coventry, UK; 2 Research and Development, University Hospitals Bristol and Weston NHS Foundation Trust, Bristol, UK; 3 Bristol Paediatric Diabetes, University Hospitals Bristol and Weston NHS Foundation Trust, Bristol, UK; 4 Bristol Medical School, University of Bristol, Bristol, UK; 5 North Somerset and South Gloucestershire Integrated Care Board, Bristol, UK; 6 Department of Paediatrics, Great Western Hospitals NHS Foundation Trust, Swindon, UK; 7 Department of Paediatrics, Royal Devon and Exeter Foundation Trust, Exeter, UK; 8 Department of Paediatrics, University Hospitals Plymouth NHS Trust, Plymouth, UK; 9 NIHR Bristol Biomedical Research Centre, University of Bristol, Bristol, UK

**Keywords:** type 1, pediatrics, glycemic control, blood glucose self-monitoring

## Abstract

**Aims:**

To assess the real-world evidence for flash glucose monitoring (Abbott FreeStyle Libre) for children with type 1 diabetes in terms of glucose control, secondary healthcare resources and costs.

**Research design and methods:**

We conducted a controlled before and after study (approximately 12 months before and after) using routinely collected health record data on children who start using flash monitors and a control population of children with self-monitoring of blood glucose (SMBG). Our population-based sample of eligible individuals using flash monitoring (n=114) and controls (n=80) aged between 4 and 18 years was drawn from four paediatric diabetes clinics (secondary care) in the South West England. Outcome measures included: glycated hemoglobin (HbA1c), frequency of BG tests; frequency of sensor scans; time in recommended glucose range; short-term complications (hypoglycemia, diabetic ketoacidosis and related illness resulting in investigation) and secondary care costs.

**Results:**

After adjustment for age, time since diagnosis, deprivation and the test modality (point of care or laboratory), the mean HbA1c reading for controls was 61.2 (mmol/mol) for the period before and 63.9 after. For individuals using flash monitoring, the adjusted mean HbA1c reading was 64.6 for the period before implementation and 63.8 after. Rates of short-term complications were low across all groups in the study. Whereas the ‘after’ flash monitoring group had substantially higher incremental costs (+£703 vs the flash monitoring ‘before’ comparison and +£841 vs contemporaneous SMBG controls), these cost differences were driven by primary care prescribing (sensor costs).

**Conclusions:**

There was some indication that flash monitoring might help young people improve the control of their diabetes but for our sample, the difference between finger-prick testing and flash monitoring was not clinically significant (HbA1c improvement <5 mmol/mol). Given the pace of technological change within diabetes, research efforts should now facilitate the real-time analysis of long-term routine data on flash and continuous glucose monitors.

**WHAT IS ALREADY KNOWN ON THIS TOPIC**

- There is growing evidence that flash monitoring is useful in helping adults achieve good glucose control.
- Evidence of effectiveness in pediatric populations has been more limited; and the resource implications of flash monitoring in these age groups are also uncertain.

**WHAT THIS STUDY ADDS**

- Our real-world study using routine data provides further indication that flash monitoring might help young people improve the control of their diabetes while also providing new evidence on the resource use implications within the National Health Service.
- For the first-generation device, we found that the increased cost of the sensor was not counterbalanced by the cost of the test strips.

**HOW THIS STUDY MIGHT AFFECT RESEARCH, PRACTICE OR POLICY**

- Given the pace of technological change within diabetes, research efforts should now facilitate the real-time analysis of long-term routine data on flash and continuous glucose monitors in young people.

## Introduction

Monitoring is essential for good glucose control. Children and young people with poor glucose control have a higher risk of developing long-term complications later in life such as kidney failure requiring dialysis or transplant, limb amputations, blindness and cardiac problems leading to early death.^1^ Additionally, in the short-term there are the risks of emergency admissions associated with hypoglycemia and diabetic ketoacidosis (DKA). In the UK, the National Health Service (NHS) spends £1 billion annually on type 1 diabetes (T1D) of which around 80% goes on treating complications.^2–4^

There are different ways to monitor blood glucose (BG); these include finger prick testing for self-monitoring of blood glucose levels (SMBG), sensors worn on the body such as flash glucose monitoring (hereafter flash monitoring) and continuous glucose monitoring (CGM) for subcutaneous glucose levels. For this study, we focused on whether flash monitoring offers improvements over and above SMBG since CGMs (which were substantially more expensive when this study was planned) were not routinely available in the UK NHS for the majority of patients until very recently.^4–6^

The only flash monitoring device available and authorized for use in the European Union is Abbott FreeStyle Libre.^5^ In adults, modeling evidence suggests flash monitoring may be cost-effective for the NHS, in part due to reduced complications.^7^ However, there is uncertainty about the impact on glucose control and healthcare resources in young people.^8 9^ Our controlled before and after study aims to consider the effectiveness, safety and resource impacts of flash monitoring compared with SMBG.

## Research design and methods

Our study aimed to examine glycemic control following the real-world implementation of flash monitoring. Overall glycemic control was measured in terms of glycated hemoglobin (HbA1c) and was captured for 12 months before and after flash monitoring (for the majority of individuals this was the first-generation device) individuals started using the device and for the equivalent period in patients who continued with SMBG. We additionally collected information on the frequency of BG tests; frequency of sensor scans; time in recommended glucose range; time in hypoglycemia (defined as measured glucose <4.0 mmol/L) and diabetes-related complications (due to hypoglycemia, DKA or other concern) resulting in attendance or admission in secondary care. Full details on the design of the study are provided elsewhere.^10 11^

### Data sources

This observational study collected data on a cohort of children who had flash monitoring initiated at any time between April 2019 and June 2020 and a further cohort of children who continued to use SMBG throughout this period (contemporaneous controls). Data were collected between October 2021 and June 2022 on flash at four NHS children’s diabetes centres (Bristol, Exeter, Swindon and Plymouth, UK), and three of these centres had capacity to provide data on control patients. Usual care for children at these centres comprises review every 3 months at multidisciplinary team clinics (routine clinics where individuals meet with pediatric specialist diabetes nurses, pediatricians, dietitians and psychologists). Eligible patients were between 4 and 18 years of age, diagnosed with T1D for at least 3 months before the January 1, 2020 and had at least 12 months of available data (to include HbA1c). All individuals interested in flash monitoring were invited to a live online training event with an Abbott FreeStyle Libre representative and one of the pediatric specialist nurses, and the families also had to provide evidence of completion for an online training module. Patients using other CGM or self-funding flash monitoring prior to April 2019 were excluded.

To avoid possible identification/re-identification of individual patients, it was a condition of the ethical approval that only clinicians with a direct care responsibility for the patients in the study centers extracted and anonymized routine data held on local center databases and (for flash monitoring and SMBG monitoring data) from the Libreview platform for analysis. We also collected baseline data from the same center database records on patient characteristics which included: sex, height, weight, partial date of birth (year and month), duration of diabetes, date commenced/stopped flash monitoring, type of insulin therapy (eg, basal/bolus regimes), concomitant pump therapy and family history of T1D. We measured area-level deprivation (from full postcode, although the latter was not stored) and ethnicity.

### Selection of patients

To minimize selection biases as far as possible, we asked centers to provide data for all eligible patients on flash monitoring. Where possible, we also asked them to provide all data for SMBG controls. Where center-specific circumstances meant that it was not possible to collect all controls (two of three centers), these centers were provided with a bespoke tool (developed in Excel) to assist random selection. For individuals using flash monitoring, the implementation date was the date the individual was judged as having started using it (based on a review of clinic letters, patient notes and Libreview platform data). For individuals using SMBG, an equivalent date was derived by randomly selecting a date (without replacement) from the observed distribution of implementation dates in the flash monitoring group. SMBG patients were excluded if they did not have a sufficient period of data available (postdiagnosis) to make them a true contemporaneous control.

### Statistical methods

Anonymized patient baseline data for the flash monitor and SMBG group were compared and, inferential testing was used to identify between-group baseline differences.

A mixed model was fitted to determine the effect of flash monitoring on log transformed HbA1c (on the natural scale), adjusting for confounding variables and including center as a random effect. The model included data from users of flash monitors and SMBG, from the before and after periods. HbA1c readings taken between 1 and 90 days after implementation or the equivalent date for the controls were excluded from the model, to allow time for flash impact HbA1c. Variables for whether or not the participant was in the flash monitoring or SMBG control group and whether or not the HbA1c reading was before or after the flash monitor implementation date (or equivalent date for controls) were included in the model; specifically, the effect of implementation was evaluated by an interaction term to test if individuals using flash monitors had different HbA1c after implementation. For this we determined a priori that clinical significance was established if we identified an increase of 5 mmol/mol or more. Using a backward stepwise regression approach, time of HbA1c reading relative to the implementation/equivalent date and the potential confounding variables age at implementation/equivalent date, sex, time since diagnosis at implementation/date, ethnicity and index of multiple deprivation (IMD) were placed in the model and were included in the final model if p<0.05.

Preliminary analysis indicated that HbA1c may have increased during the period April–June 2020, which coincided with the initial COVID-19 lockdown where general practitioners in some areas were asked to take blood samples for laboratory testing, as opposed to the usual point-of-care testing which uses a different test assay. To avoid possible bias associated with the measurement technique, a variable for the HbA1c test modality was also included in the backward stepwise modeling.

The final mixed model with center as a random effect, adjusted for the statistically significant variables, age (<12, 12–15, 16+ years), time since diagnosis (<2 years, 2+ years), IMD decile (categorized as deciles 1–4 vs 5–10), test modality and the flash monitor/‘after’ period interaction term.

### Methods for diabetes-related complications, resource use and costs

We adjusted all resources (test strips, sensors and contacts/admissions for diabetes complications) for the variable length of follow-up using an incidence rate approach (ie, resource units used per person year). We subsequently valued resource data associated with loss of glucose control (ie, hypoglycemic episodes, DKA events and other diabetes complications) using an NHS secondary care perspective (2019/20 price year) for the before and after periods for both groups of patients (online supplemental table S1).^12^

10.1136/bmjdrc-2023-003561.supp1 Supplementary data

Our before and after study analysis estimates primary care prescribing costs associated with BG testing strips and lancets based on recorded usage (this was estimated at patient level using the number of recorded BG measurements in the individual’s medical record).^5^ The brand of BG testing lancet/ strips most frequently used was obtained via application to a single clinical commissioning group (now integrated care board) and valued using NHS sources.^13^ ^14^ Assumptions for resource items are further outlined in online supplemental table S1.

## Results

Data were collected on 114 flash monitor users and 80 SMBG controls; from 4 centers for the flash monitoring group and from 3 of the centers for the control group. A comparison of the baseline characteristics of the groups showed that the control group had more male participants (56% vs 42%, p=0.0590), were taller (median height 161 cm vs 153 cm, p=0.0526) and heavier (median weight 55 kg vs 48 kg, p=0.0455). Groups had similar HbA1c at implementation (table 1). In line with the advice of the STROBE guidelines for reporting observational studies (online supplemental file 4), we provide a Study Flow (online supplemental file 5).

10.1136/bmjdrc-2023-003561.supp4 Supplementary data

10.1136/bmjdrc-2023-003561.supp5 Supplementary data

**Table 1:** Baseline characteristics

	Flash monitoring group (n=114)	SMBG control group (n=80)	P value*
Center, no (%)			
Bristol	81 (71%)	60 (75%)	
Exeter	13 (11%)	0	
Plymouth	8 (7%)	8 (10%)	
Swindon	12 (11%)	12 (15%)	
Age,† median (IQR)	12 (10, 14)	13 (11, 15)	0.1793
Male, no (%)	48 (42%)	45 (56%)	0.059
Ethnicity, n (%)			
White	94 (82%)	67 (84%)	0.766
Asian	6 (5.3%)	2 (2.5%)	
Black	10 (8.8%)	6 (7.5%)	
Mixed	3 (2.6%)	3 (3.8%)	
Other	1 (0.9%)	2 (2.5%)	
IMD decile, n (%)	N=112	N=79	0.1662
1, 2	32 (29%)	22 (28%)	
3, 4	16 (14%)	6 (7.6%)	
5, 6	25 (22%)	17 (22%)	
7, 8	24 (21%)	16 (20%)	
9, 10	15 (13%)	18 (23%)	
Celiac disease, no (%)	3 (2.6%)	1 (1.3%)	0.644
Hypothyroidism, no (%)	6 (5.3%)	3 (3.8%)	0.739
Family history of diabetes, no (%)	19 (17%)	9 (11%)	0.309
Eating disorder, no (%)	1 (0.9%)	1 (1.3%)	1.000
Height,† median (IQR), cm	153 (138, 165)	161 (142, 172)	0.0526
Weight,† median (IQR), kg	48 (34, 59)	55 (37, 68)	0.0455
Time since diagnosis (years),† median (IQR)	3.2 (1.3, 5.6)	4.3 (1.4, 7.4)	0.0926
Type of insulin therapy, no (%)†			
Mutliple daily injection regime	95 (83%)	66 (83%)	1.000
Pump therapy ±	12 (11%)	3 (3.8%)	0.104
Unknown	7 (6%)	11 (14%)	
Started Libre2, n (%)	7 (6.1%)		
HbA1c (mmol/mol),† median (IQR)	62 (57, 72)	61 (53, 72)	0.2751

IQR (lower quartile, upper quartile).

*P values obtained by Mann-Whitney U test or Fisher’s exact test. IMD decile calculated from the postcode and corresponds to the LSOA that each postcode falls within±those on pump therapy were more likely to be prescribed alternate CGM devices than Libre, and were thus not eligible to be included in this study. Libre2=second-generation flash monitoring device, with built-in alarms.

†At flash monitoring implementation date for the ‘flash’ group and at the equivalent date for the controls or for weight, height and HbA1c, the value immediately prior to flash implementation/equivalent date.

CGM, continuous glucose monitoring; HbA1c, glycated hemoglobin; IMD, index of multiple deprivation; LSOA, lower-layer super output area; SMBG, self-monitoring of blood glucose.

### Preliminary descriptive analysis

To explore the impact of national restrictions following the pandemic, we additionally explored HbA1c by calendar time and modality. Comparison of laboratory versus point-of-care testing identified that laboratory-based tests, which use a different assay, were on average higher (observed median 66 mmol/mol compared with 63 mmol/mol, an increase of 3 mmol/mol).

### HbA1c and NICE targets

**Figure 1:**
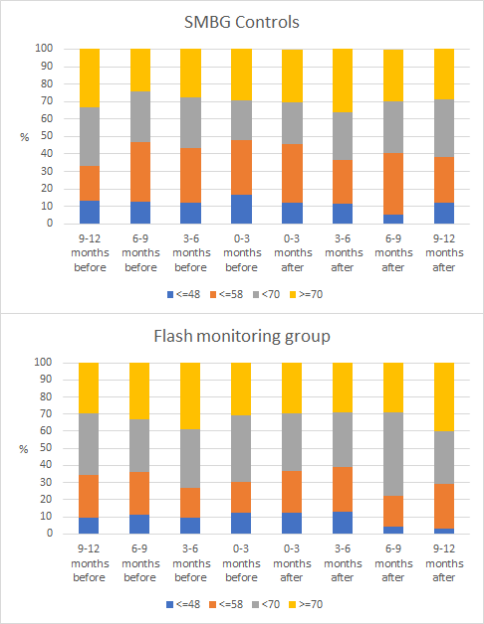
Glycated hemoglobin (HbA1c) according to HbA1c target ranges by quarter for individuals using flash and self-monitoring of blood glucose (SMBG) controls.

The proportion of individuals achieving the NICE target of ≤48 mmol/mol was below 20% at all timepoints for both flash monitor and SMBG controls (figure 1). The proportion of individuals above the NICE target of >70 mmol/mol ranged from 29% to 39% before implementation for the flash monitor group, compared with 24% to 33% for controls. After implementation, the equivalent ranges for >70 mmol/mol were 29%–40% for flash monitoring and 29%–36% for controls.

### Adjustment of HbA1c for confounding

**Figure 2:**
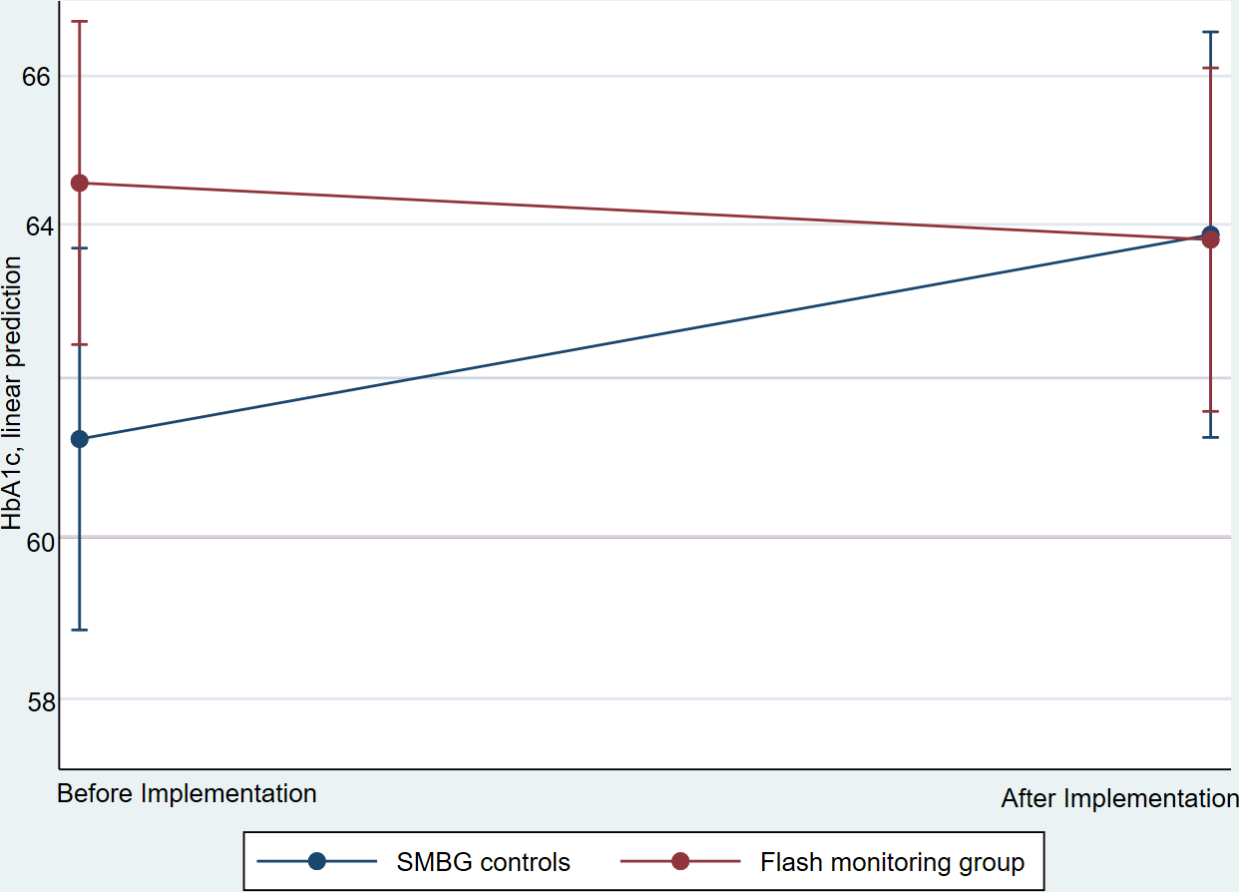
Predictive margins of glycated hemoglobin (HbA1c) before and after implementation of flash monitoring. SMBG, self-monitoring of blood glucose.

Figure 2 shows the predictive margins from the mixed model of HbA1c before and after implementation for individuals using flash monitors and controls. The adjusted mean HbA1c reading was 61.2 for the controls in the period before the equivalent date, and 63.9 after, a 4.3% (95% CI 1.7% to 7.0%) increase. For individuals using flash monitors, the adjusted mean HbA1c reading was 64.6 in the period before implementation and 63.8 after, a decrease of (−)1.2%. (95% CI −3.3% to 1.0%) decrease (figure 2). Detailed results from the final mixed model are shown in online supplemental table S2.

10.1136/bmjdrc-2023-003561.supp2 Supplementary data

### Other outcomes relating to glucose control

There was no evidence of a difference in average BG (mmol/L) between flash monitor users and controls over the study duration (supporting information in online supplemental table S3).

10.1136/bmjdrc-2023-003561.supp3 Supplementary data

For individuals using flash monitors with available data on sensor use (n=106), the median % time achieved in range in the 2-week period preceding their multidisciplinary team clinic was 46% (IQR 34%–55%); the corresponding time in hypoglycemia was 5.5% (IQR 2.6%–8.8%).

### Sensor scans and blood glucose test frequency

Overall, control individuals tested slightly less frequently before implementation compared with individuals using flash monitoring. Both groups reduced BG testing after -implementation using 4.8 (0.5 fewer compared with same group in the before period) and 4.1 (1.9 fewer compared with same group in the before period) test strips in the control and flash groups, respectively. The median number of daily scans for individuals using flash monitors was 6.7 (IQR 4.3, 10) (n=103). The majority of individuals in the flash monitoring group used the first-generation device; seven individuals (6.1%) were recorded as having switched to a new generation flash monitor with built-in alarms (Libre2) in the study period.

### Diabetes-related complications, healthcare resource and costs

The proportion of individuals where the clinician was able to confirm data on complications was at least 78% for each of the four groups (SMBG before, SMBG after, flash monitoring before, flash monitoring after). The observed incident rates of diabetes-related complications were extremely low for all patients. While there were five admissions for hypoglycemia in the flash group after implementation, the total number of admissions (which includes DKA and other admissions) was similar (table 2).

**Table 2:** Complications observed before/after implementation (or equivalent period, for SMBG controls)

	Resource units observed before and after implementation. Frequency (number of patients affected)				Incidence rate (standardized per person year)			
	SMBG before	SMBG after	Flash monitoring before	Flash monitoring after	SMBG before	SMBG after	Flash monitoring before	Flash monitoring after
	N=80		N=80		N=114		N=114	
Outpatient	6	9	2	4	0.087	0.131	0.022	0.037
ED (diabetes related) and discharge	2	2	2	2	0.029	0.029	0.022	0.018
ED leading to admission	6	1	10	10	0.087	0.015	0.110	0.092
Admission—any diabetes	10	4 (n=3)*	13	14 (n=12)*	0.146	0.058	0.143	0.129
Admission—hypo	3	0	1	5	0.044	0.000	0.011	0.046
Admission—DKA	1	1	4	1	0.015	0.015	0.044	0.009
Admission—other diabetes	6	3	8	8	0.087	0.044	0.088	0.074

*One or more patients had multiple events.

DKA, diabetic ketoacidosis; ED, emergency department; SMBG, self-monitoring of blood glucose.

Table 3 shows that primary and secondary care costs in the two before groups were similar. After implementation, flash monitoring was more costly at £1703.50 by an incremental cost of £909.32 (BG contemporaneous controls) and £750.43 (the before comparison using flash monitoring). After adjustment for potential confounders, and center effects, the incremental cost differences reduced slightly (£840.50 and £703.12, respectively).

**Table 3:** Resource use, and costs before and after implementation

Resource use and costs	SMBG before (n=71)	SMBG after (n=69)	Flash monitoring before (n=102)	Flash monitoring after (n=89)
Resource use				
Mean daily BG testing strips and lancets used (SD)*	5.48	4.80	5.97	4.30
Mean daily scans (SD)*	NA	NA	NA	6.70
First-generation/Second-generation sensors (per annum)	NA	NA	NA	13
Costs				
Total primary care prescribing†	£706.59	£619.05	£770.18	£1468.43
Secondary care cost	£242.88	£175.14	£182.89	£235.07
Total NHS cost‡	£949.47	£794.18	£953.07	£1703.50
Adjusted NHS cost and 95% CI§	£960.81 (£793.25 to £1128.37)	£822.94 (£655.71 to 990.18)	£960.32 (£820.22 to 1100.41)	£1663.44 (£1505.68 to £1821.21)

*Complete case analysis. Not all individuals contributed sufficient data in the 2-week period preceding the clinic date to calculate a mean number of scans.

†Primary care prescribing estimated based on BG test strip and flash monitoring sensor usage, based on mean number of readings in preceding 2-week period.

‡Units unadjusted other than for variable follow-up; costs are mean per person year costs based on individual patient data.

§Linear prediction via a two-level mixed model adjusting for variance difference between patients in terms of IMD, time since diagnosis, age, test modality (point of care or laboratory).

IMD, index of multiple deprivation; NA, not available; NHS, National Health Service; SMBG, self-monitoring of blood glucose.

## Conclusions

There was some indication that flash monitoring might help young people improve the control of their diabetes but for our sample, the difference between finger-prick testing and flash monitoring was not deemed clinically significant (HbA1c improvement <5 mmol/mol). There was no evidence of a difference in average BG levels and the overall rate of diabetes-related complications were low across all groups and similar.

From a combined primary and secondary care perspective, the first-generation flash device was more costly than SMBG, with the incremental cost difference observed being similar to the outlay associated with a flash sensor (£900 for the 2019/20 price year).

A strength of this study is that it explored the rollout of a new technology into clinical practice. As there was no requirement for individual patient consent, the population may be more generalizable than the self-selected population who consent for clinical trials. Notwithstanding this, there is still the possibility of selection bias with the naturalistic design because there may be unidentified differences between the flash and control groups which were not controlled for. For instance, initial UK NHS prescribing guidance suggested that flash monitoring should be made available for young people needing to BG test at least 8 times a day.^15^ It is possible that this in part was a cost-containment strategy to restrict eligibility to children and families who use large numbers of BG tests. However, we did not detect a difference in the median test frequency in the control and flash monitoring groups prior to implementation. Additionally, it is possible that the retrospective study of complications and costs is affected by information biases due to missing data, although overall completeness was high at over 86%.

A challenge was that our study data collection period overlaps with the COVID-19-SARS-CoV-2 pandemic and a period of national restrictions including lockdowns, which may have had a dual effect both on glucose control, and the measurement of HbA1c (which affected the frequency of face-to-face reviews and therefore point-of-care HbA1c tests). However, as far as possible we attempted to control for this in our adjusted analyses by controlling for the test modality. The pandemic also affected our ability to collect data on any SMBG controls from one of the centers, as our ethical approval meant we were reliant on direct-care clinicians extracting and anonymizing individual patient data. While incomplete outcome data bring a risk of bias, the missing controls related to one of the smaller centers, which provided 11.4% of individuals using flash monitors, and would therefore be unlikely to overturn study results. While the pandemic might also have meant that some routine HbA1c tests were not carried out, a benefit of a mixed model is the flexibility and efficiency in handling missing and unequal measurements per subject.

A further challenge was that the new generation flash monitor (Libre2) was approved for use within the NHS in November 2020, meaning that seven individuals had the sensor prescribed during the study period. This was too small a sample with insufficient follow-up to report separately and limits the generalizability of our results.

Our economic analysis was based on recorded BG test frequencies within the study. It is possible that usage may differ from actual prescribing, and therefore our real-world assessment of flash monitoring might be somewhat conservative about prescribing costs, although the measurement bias should affect both groups similarly. However, the recorded BG test frequencies for individuals using FLASH collected in this study reflect the measurements needed to ensure carbohydrate counting and insulin dosage administration was calculated accurately for meals and snacks. With the advance of new generation flash monitors with built-in alarms and app technology such as the ‘My Life’ app, these measurements using traditional BG sampling are no longer required. This means that we cannot extrapolate these results to the present clinical scenario without caveat.

This was a partial economic evaluation that did not quantify the improvements in quality of life (QoL) associated with using flash monitoring which are evident from interviews we carried out with young people and their parents within the qualitative study in helping young people ‘get on with their lives’ and reducing parent/carer worry.^16^ Additionally, collection of QoL data would enable future long-term modeling, which to date has primarily been limited to adult populations.^7 17–19^ We also note that the study was limited to a single region within England; elsewhere, a population-based national assessment of costs relating to a UK adult population at a similar point in time found evidence of cost-offsetting in terms of hospital admissions.^20^

In the UK, NHS England made the decision to reimburse the cost of flash where people with T1D met eligibility criteria in 2019 while they collected more evidence.^15 21^ The reduced requirement for BG testing with new generation alarmed devices may offer improvements in glucose control for individuals^22^ while also offering value for money for the NHS but this needs comparison now with all available real-time CGM devices, including in adults with Dexcom ONE Real Time-CGM where the NHS has brokered a cut-price pay deal.^23^ To assist with this, we now need long-term routine data—with a sample size sufficiently powered to assess the impact on complications—which examines the cost-utility of flash and continuous glucose monitors. This can be facilitated by: (1) an efficient approach to data capture such that anonymized sharing of center records is achieved without the employment of direct-care staff; (2) routine recording of health-related QoL at clinic appointments perhaps annually; (3) making sure studies follow-up teenagers into early adulthood as they transition to adult services and glucose management can falter and (4) reducing inequalities in access to technology to enable individuals at high risk (HbA1c above target) to benefit.

## Data Availability

Data may be obtained from a third party and are not publicly available.
