# Supplementary material for: Implementation of flash glucose monitoring in four pediatric diabetes clinics: controlled before and after study to produce real-world evidence of patient benefit
Source: BMJ Open Diabetes Res Care. 2023 Aug 28;11(4):e003561. doi: 10.1136/bmjdrc-2023-003561 (PMC10462967; doi:10.1136/bmjdrc-2023-003561)
Supplement: Supplementary data [file bmjdrc-2023-003561supp001.pdf]

Supplementary appendix

S1 Table: Unit costs

Resource	Unit cost, £	Source and assumption
BG monitor	Not included	For patients with already diagnosed diabetes, no change in BG monitor provision
BG testing strips (pack of 50)	16.21	Based on most commonly used BG testing strip, BNF 79. (14)
BG lancet (pack of 100)	2.89	Based on most commonly prescribed multi-device lancet (13)
Flash Reader (first/ second gen)	Free	Free supply by Abbott (to all NHS patients)
Sensor (first/second gen)	35.00	FreeStyle Libre sensor, Abbott laboratories, BNF 79. (12) Cost per year (£913.13) based on a year's supply, where sensor is changed every 14 days.
Outpatient appointment (non-routine)	258	Consultant-led paediatric endocrinology, 2019-20 National Schedule of NHS costs.(12)
ED and discharge	151	Mean weighted cost based on activity of all non-admitted HRG for Emergency Medicine Cat 1 or 2 investigation and 1 or 2 treatment 2019-20 National Schedule of NHS costs. (12)
ED leading to admission	243	As above, for all admitted cases
Admission – hypo	2,078	Mean cost based on activity of all HRGs 2019-20 National Schedule of NHS costs. Paediatric Diabetes Mellitus, with Ketoacidosis or Coma. (12)
Admission – DKA	2,044	As above, Paediatric Diabetes Mellitus, with Ketoacidosis or Coma
Admission – other diabetes related	986	Non-elective short stay paediatric diabetes mellitus, CC Score of 0. (12)
