# Supplementary figures and images for: Implementation of flash glucose monitoring in four pediatric diabetes clinics: controlled before and after study to produce real-world evidence of patient benefit
Source: BMJ Open Diabetes Res Care. 2023 Aug 28;11(4):e003561. doi: 10.1136/bmjdrc-2023-003561 (PMC10462967; doi:10.1136/bmjdrc-2023-003561)

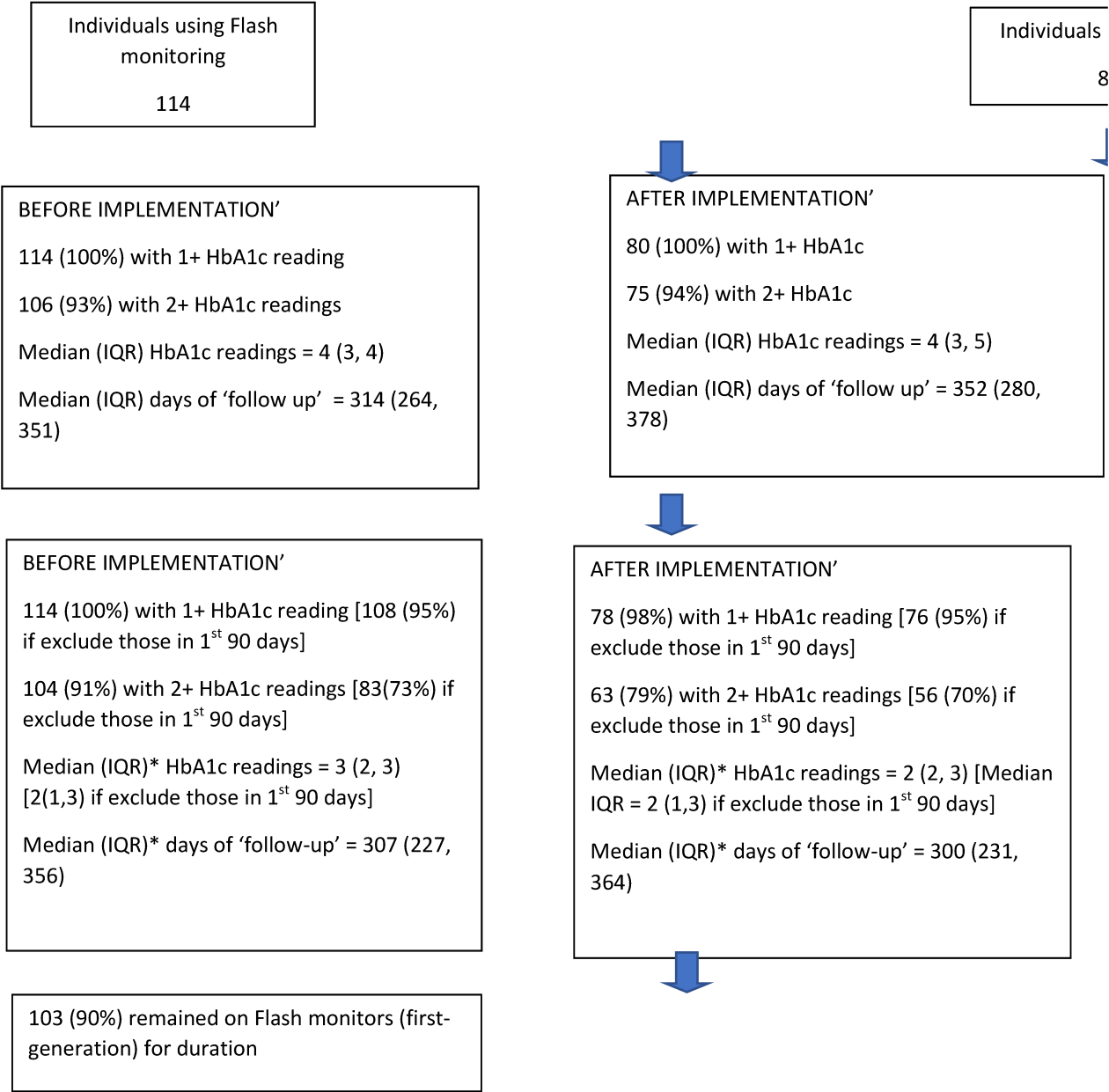

Supplementary material: Study flow

Supplement: Supplementary data [file bmjdrc-2023-003561supp005.pdf]
