# Supplementary material for: Implementation of flash glucose monitoring in four pediatric diabetes clinics: controlled before and after study to produce real-world evidence of patient benefit
Source: BMJ Open Diabetes Res Care. 2023 Aug 28;11(4):e003561. doi: 10.1136/bmjdrc-2023-003561 (PMC10462967; doi:10.1136/bmjdrc-2023-003561)
Supplement: Supplementary data [file bmjdrc-2023-003561supp002.pdf]

S2 Table: Mixed model.

	Unstandardised coefficient		% change in HbA1c per one unit increase in predictor		
	β	95% Confidence Interval		z	P-value
After Implementation or equivalent period (vs'before')	0.042	(0.017, 0.068)	4.3	3.3	<0.001
Used Flash monitor (vs SMBG)	0.053	(0.0012, 0.11)	5.5	2.0	0.045
Interaction 'after' period/Flash monitoring	-0.054	(-0.087, -0.021)	-5.3	-3.2	<0.001
Age (*) 12-15	0.082	(0.027, 0.14)	8.5	2.9	0.003
Age (*) 16+	0.18	(0.082, 0.27)	19	3.7	<0.001
2+ (*) years since diagnosis	0.15	(0.091, 0.20)	16	5.2	<0.001
HbA1c measured at Lab	0.053	(0.024, 0.082)	5.5	3.7	<0.001
IMD decile 1-4 (vs 5-10)	0.061	(0.0096, 0.11)	6.3	2.3	0.02
Constant	3.92	(3.86, 3.98)		119	<0.001
Random Effects Parameters	Estimate				
Centre Variance	0.00	(0.00, 0.019)			
Patient Variance	0.028	(0.022, 0.035)			
Residual Variance	0.015	(0.014, 0.017)			

(*) At implementation date/ equivalent date

Dependent variable is $\log_e$(HbA1c). Model coefficients are on the natural log-scale; results were back-transformed, that is exponentiated, to obtain percentage differences in HbA1c.

After adjustment, for controls the natural log of HbA1c increased by 0.042 from before to after periods, equivalent to a 4.3% increase in HbA1c ($e^{0.042}$). For the flash monitoring group, the natural log of HbA1c decreased by the natural log 0.012 (=0.042-0.054) from before to after periods. This is equivalent to a decrease of (-) 1.2% in HbA1c ($1-e^{(0.042-0.054)}$).
