# Supplementary material for: Implementation of flash glucose monitoring in four pediatric diabetes clinics: controlled before and after study to produce real-world evidence of patient benefit
Source: BMJ Open Diabetes Res Care. 2023 Aug 28;11(4):e003561. doi: 10.1136/bmjdrc-2023-003561 (PMC10462967; doi:10.1136/bmjdrc-2023-003561)
Supplement: Supplementary data [file bmjdrc-2023-003561supp003.pdf]

S3 Table: Summary measures of glucose control and process measures relating to use

**Summary Measures**		n	
2 week blood glucose (mg/dL); Median (IQR)	Control before	69	9.0 (8.0, 11.3)
	Control after	61	9.5 (8.3, 11.3)
	Flash monitoring before	87	9.0 (8.0, 10.6)
	Flash monitoring after	104	10.1 (9.0, 12.0)
% time in range[1]; Median (IQR)		106	46% (34%, 55%)
% time in hypoglycaemia[1]; Median (IQR)		104	5.5% (2.6%, 8.8%)
**Process measures**			
Mean number of daily scans[1]; Median (IQR)		103	6.7 (4.3, 10.0)
No. of daily BG tests; Median daily (IQR)	Control before	71	5.3 (3.8, 7.1)
	Control after	69	4.8 (3.5, 6.1)
	Flash before	102	6.0 (3.8, 8.0)
	Flash after	88	4.1 (2.4, 6.4)

1 Flash monitor users only

IQR = Interquartile Range (Lower quartile, upper quartile)
Quarterly readings were collected relating to the two week's prior. While we report the sample median, our healthcare professionals reported individual means for clinical outcomes based on readings observed over a two-week period immediately prior to their clinic appointment (% time in range, % time in hypoglycaemia, and BG level).
